# Intervention effects of high-intensity interval exercise and moderate-intensity continuous exercise on executive functions in obese adolescents with low socioeconomic status

**DOI:** 10.3389/fpsyg.2026.1621897

**Published:** 2026-01-28

**Authors:** Xiaotao Wang

**Affiliations:** Hainan Police College, Haikou, China

**Keywords:** executive function, high-intensity interval exercise, low socioeconomic status, moderate-intensity continuous exercise, obese adolescents

## Abstract

**Objective:**

This study aimed to compare the intervention effects of high-intensity interval exercise (HIIE) and moderate-intensity continuous exercise (MICE) on the executive functions of obese adolescents with low socioeconomic status (SES).

**Methods:**

A total of 84 obese adolescents aged 12 ~ 15 years with low SES were selected and randomly divided into three groups: the HIIE group, the MICE group, and the control group, with 28 participants in each group. The HIIE group underwent 12 weeks of combined aerobic and anaerobic training, performed 3 times per week for 60 min each session. The MICE group conducted 12 weeks of outdoor jogging, 3 times per week for 60 min each session. The control group maintained their normal daily life and academic activities. The GO/NOGO task, 2-back task, and More-odd shifting task were used to assess inhibitory control, working memory, and cognitive flexibility, respectively. Meanwhile, body mass index (BMI) and heart rate were monitored throughout the study.

**Results:**

After the intervention, both the HIIE group and the MICE group showed significant improvements in inhibitory control, working memory, and cognitive flexibility (all *p* < 0.05). Specifically, the MICE group exhibited a more significant increase in the accuracy rate of inhibitory control (*F* = 3.721, *p* = 0.029, *η*^2^ = 0.100), while the HIIE group demonstrated a more pronounced reduction in the reaction time of cognitive flexibility (*F* = 5.343, *p* = 0.007, *η*^2^ = 0.138). Regarding BMI, the HIIE group had a significantly lower BMI than both the MICE group (*p* = 0.027) and the control group (*p* < 0.001), and the MICE group also had a significantly lower BMI than the control group (*p* = 0.008).

**Conclusion:**

Both HIIE and MICE can effectively improve the executive functions and BMI of obese adolescents with low SES, but they exert selective promoting effects. HIIE is more advantageous in improving BMI and reducing the reaction time of cognitive flexibility, while MICE is superior in enhancing the accuracy rate of inhibitory control.

## 1 Introduction

Obesity has become a severe public health issue globally (James, 2018). Particularly, the increasing prevalence of obesity among adolescents has drawn increasing attention from all sectors of society (Bibiloni et al., 2013). According to data from the World Health Organization (WHO), the obesity rate among adolescents has been on the rise over the past few decades (Spinelli et al., 2021). In China, with the rapid development of the social economy and the shift in lifestyle, the problem of adolescent obesity has also become more prominent (Liang and Qi, 2020). Relevant research statistics show that the overweight rate among Chinese children and adolescents has risen to 12.2%, and the obesity rate has reached as high as 7.1% (National Health Commission, 2020).

Socioeconomic status (SES) is closely linked to adolescent obesity. Although current research findings are not consistent, some studies (Zhang et al., 2023; Zhao et al., 2024) suggest that higher SES is a major factor in adolescent obesity, while others (Fradkin et al., 2015; Frederick et al., 2014) indicate that lower SES is the main factor. However, it is undeniable that adolescent obesity in China is spreading to rural areas with lower SES (Dang et al., 2025; Dong et al., 2019). Moreover, some studies (Vieweg et al., 2007; Zhang et al., 2023; Zhao et al., 2024) have found that adolescents with low SES are increasingly becoming a high-risk group for obesity. Adolescents with low SES often face more health risk factors, such as unhealthy dietary environments, lack of opportunities for physical activity, and limited access to health resources (Gautam et al., 2023; Schreier and Chen, 2013). In terms of diet, low-SES families, due to economic constraints, tend to choose foods that are cheap but high in energy and low in nutritional value (Baumann et al., 2019). These foods are usually rich in sugar, salt, and saturated fats, but lack vitamins, minerals, and dietary fiber. In terms of lifestyle, low-SES communities may lack safe places and facilities for physical activity, and have inconvenient public transportation, which leads adolescents to rely more on sedentary activities, such as watching TV and playing video games (Moore et al., 2008). Long-term unhealthy diets and lifestyles make adolescents with low SES more prone to obesity.

Obesity not only affects the physical appearance of adolescents but also has many negative impacts on their physical and mental health. For example, obesity is closely related to an increased risk of developing various chronic diseases such as hypertension (Flynn and Falkner, 2011), cardiovascular diseases (Sommer and Twig, 2018); it is also closely associated with psychological problems like depression (Quek et al., 2017), anxiety (Lindberg et al., 2020), and may even have adverse effects on their academic performance and future career development (Harris, 2019; Ma et al., 2020). Executive function is a psychological process in which an individual consciously controls thoughts and actions in the process of achieving goals, including core components such as inhibitory control, working memory, and cognitive flexibility (Shi et al., 2022). Executive function plays a crucial role in adolescents’ learning, social interaction, and daily life (Moriguchi, 2014). Good executive function helps adolescents focus better, solve problems, make plans, and regulate emotions (Shi et al., 2022). Existing studies (Lokken et al., 2009; Meo et al., 2019) have shown that there is a link between obesity and impaired executive function in adolescents. Obesity may affect executive function through various physiological and psychological mechanisms. For example, chronic inflammatory responses, oxidative stress, and insulin resistance caused by obesity may have adverse effects on brain structure and function (Ghowsi et al., 2021), thereby impairing executive function. In addition, due to poor eating habits and a sedentary lifestyle, obese adolescents may affect the development and function of the brain, leading to a decline in executive function (Dutra et al., 2016). Thus, it is of great importance to find a suitable method to improve the executive function of obese adolescents.

Physical exercise, as a non-pharmacological intervention, is widely recognized as an effective method to improve the health status of obese adolescents. Exercise can have positive effects on the physical and psychological well-being of obese adolescents through various pathways, such as increasing energy expenditure, improving metabolic function, and regulating neurotransmitters. A large number of studies (Cerda-Vega et al., 2024; Chen et al., 2016; Liu et al., 2018) have shown that physical exercise can serve as an important means of improving the executive function of obese adolescents. High-intensity interval exercise (HIIE) and moderate-intensity continuous exercise (MICE) are two common forms of exercise, which differ in terms of exercise intensity, exercise pattern, and energy expenditure, and may have different impacts on the executive function of obese adolescents (Zhang et al., 2022). Relevant studies (Bunce and Murden, 2006; Weinstein et al., 2012) have found that the higher the aerobic exercise capacity, the better the performance in executive function. Liu et al. (2020) have also shown that MICE intervention can effectively improve the executive function of adolescents. In addition, HIIE, with its characteristics of diverse movement combinations, high efficiency, and strong rhythm, has gradually attracted attention. Many studies (Leahy et al., 2020; Reyes-Amigo et al., 2022) have also found that HIIE can effectively enhance the executive function of adolescents. Moreover, relevant studies have compared the effects of HIIE and MICE interventions on the executive function of adult populations and adolescent populations, but the results of these studies are not consistent. For example, Wu et al. (2023) found that both HIIE and MICE can improve inhibitory control in healthy populations, and there is no significant difference in their improvement effects. However, Tian et al. (2021a, 2021b) found that HIIE is more effective than MICE in improving inhibitory control and cognitive flexibility. Some other studies (Jia, 2022; Shang, 2022) have found that MICE have better intervention effects on self-control and working memory than HIIE. In addition, there is currently no research focusing on the low SES obese adolescent population, and it is unclear how HIIE and MICE interventions affect the executive function of low SES obese adolescents.

Based on this, the present study aims to experimentally compare the intervention effects of HIIE and MICE on the executive function of low SES obese adolescents, analyze the advantages and applicability of the two types of exercise, and provide a scientific exercise plan to promote the cognitive development of low SES obese adolescents. Additionally, the findings of this study can offer a scientific basis for government departments to formulate relevant public health policies, promote the implementation of exercise intervention programs and health promotion plans targeting low SES obese adolescents, facilitate the healthy development of the adolescent population, and alleviate the social medical burden.

## 2 Methods

### 2.1 Participants

In this study, the sample size was calculated using G*Power 3.1.9.7 software. Referring to the meta-analysis by Hu et al. (2021) on the effects of HIIE on executive function in adolescents, the effect size (*ES*) was extracted as 0.38. The significance level (*α*) was set at 0.05, and the power (1-*β*) was set at 0.8. The calculation indicated that a total of 49 participants were needed, meaning that 25 participants were required for each of the HIIE and control groups. Additionally, another 25 participants were recruited for the MICE group to compare the intervention effects of HIIE and MICE on executive function in adolescents. Therefore, a total of 75 participants were needed for this study. The participants in this study were low-SES obese adolescents. Firstly, communication was established with a junior high school in a rural area to screen for obese adolescents. Secondly, the SES of the screened obese adolescents was assessed to identify those with low SES. A total of 120 participants were ultimately recruited for the study. To ensure the accuracy of the study results, the following inclusion criteria for participants were established in this study:

(1) Adolescents aged between 12 and 15 years old. (2) Using the Children and Adolescents BMI Calculator^1^, the BMI index of each adolescent is calculated based on their height, weight, age, and gender, and those identified as obese are included. This calculator is developed based on the BMI screening criteria for overweight and obesity among Chinese students (Ji, 2004) and shows no discrepancy from manual calculations. (3) The MacArthur Scale of Subjective Socioeconomic Status is used to evaluate the SES of adolescents (Galvan et al., 2023), and those with an SES score below 5 are included. (4) Adolescents with no history of major diseases, such as cardiovascular diseases, respiratory diseases, or neurological diseases, to ensure the safety of exercise interventions. (5) Adolescents who have no medical contraindications to exercise, such as unrecovered joint injuries or severe musculoskeletal disorders, and who are able to engage in regular physical activity without restrictions. (6) Adolescents with normal vision or corrected vision, and no color blindness or color weakness. (7) Adolescents with normal intelligence and mental status. (8) If a participant is absent from exercise sessions more than twice, they will be excluded from the data analysis.

After preliminary screening and assessment, 36 adolescents who did not meet the criteria were excluded, resulting in a total of 84 participants for the study. These participants were randomly assigned to three groups: the HIIE group, the MICE group, and the control group, with 28 participants in each group. Randomization was achieved using a computer-generated random number method to ensure the randomness and balance of group assignment, thereby minimizing the impact of intergroup differences on the study results. This study was approved by the Scientific Research Ethics Committee of Hainan Police College. All participants provided informed consent, and both the participants and their guardians were fully informed about the purpose, methods, procedures, and potential risks of the study, and they voluntarily agreed to participate in this research.

### 2.2 The design of the intervention program

Two coaches are assigned to guide the HIIE group and the MICE group, respectively, during the exercise to ensure the accuracy of the intervention measures. The training programs of the HIIE group and the MICE group are significantly different. Having separate coaches for each group can ensure that the training of each group is carried out strictly in accordance with the established program, guarantee the accuracy of the intervention measures, and reduce the deviation in the implementation of the training program caused by human factors. In addition, it can also avoid cross-interference and eliminate the influence of the coaches’ personal preferences. If the same coach is in charge of guiding both groups, there may be an inadvertent infiltration of the training methods or concepts of the HIIE group into the MICE group, or vice versa, thus affecting the independence and purity of the training of the two groups. However, having two coaches guide the two groups, respectively, can effectively avoid such cross-interference, keep a clear boundary for the training interventions of the two groups, reduce the influence of confounding factors on the experimental results, and ensure that the experiment can accurately evaluate the respective effects of the two training methods, namely HIIE and MICE. The specific intervention plans for HIIE and MICE are detailed as follows:

#### 2.2.1 HIIE group

The HIIE group’s design combines aerobic and anaerobic exercises, including sprinting, skipping rope, burpees, etc. The sprinting is carried out on the straight track of a standard 400 m athletics field, with each sprint covering a distance of 100 m. The speed is required to reach over 90% of the individual’s maximum speed, rapidly increasing the heart rate to 85 to 95% of the maximum heart rate. This intensity can effectively stimulate the cardiopulmonary function and the anaerobic metabolism system. The skipping rope exercise is performed in the way of fast double-foot jumping, lasting for 1 min each time, and the number of skips per minute should reach 180 to 200 times, which can also bring the body into a high-intensity exercise state. For the burpees, the movements are required to be standardized, including movements such as squats, push-ups, and jumps. Each burpee session lasts for 45 s, quickly consuming energy in a short time and increasing the exercise intensity.

Each HIIE group workout session has a total duration of 60 min, including a 5-min warm-up session, a 50-min HIIE session, and a 5-min relaxation and stretching session. The warm-up mainly involves activities such as slow walking and dynamic stretching to fully activate all the joints and muscles of the body, increase body temperature and heart rate, prepare for the upcoming high-intensity exercise, and reduce the risk of sports injuries. The HIIE session mainly consists of multiple sets of circular training according to different sports items. For example, first, do a 100 m sprint, and then immediately follow it with 2 min of slow walking or marching in place for rest, allowing the heart rate to decrease but still remain at a relatively high level; then do 1 min of fast skipping rope and rest for 1 min; then do 45 s of burpees and rest for 1 min, and so on for 6 to 8 sets in a cycle. The relaxation session mainly involves static stretching to relax the muscles, relieve post-exercise fatigue, and promote physical recovery.

A systematic review and meta-analysis by Liu et al. (2024) revealed that HIIE lasting more than 8 weeks could effectively enhance participants’ executive function. On this basis, the present study adopted the protocol of Liu et al. (2024) and determined the intervention duration as 12 weeks by considering the basic conditions of the participating schools and students. Additionally, the training was scheduled 3 times per week, specifically on Mondays, Wednesdays and Fridays, to allow sufficient recovery time for the body and avoid fatigue and sports injuries caused by overtraining. During the exercise process, professional sports coaches are assigned to provide guidance and supervision to ensure that the teenagers perform the movements correctly and avoid sports injuries caused by improper movements. The coaches will adjust the exercise intensity and rest time in a timely manner according to each teenager’s physical condition and exercise performance. For example, if it is found that a certain teenager’s heart rate recovers too slowly after the sprint or shows signs of excessive fatigue, the rest time will be appropriately extended or the intensity of the next set of exercises will be reduced.

#### 2.2.2 MICE group

The MICE group mainly engages in outdoor jogging. The sports venue is the campus athletics field, and the running speed is adjusted according to the physical conditions and sports abilities of the teenagers, maintaining a level that can keep the heart rate at 60 to 70% of the maximum heart rate. Each MICT group workout session has a total duration of 60 min, including a 5-min warm-up session, a 50-min MICE session, and a 5-min relaxation and stretching session. The warm-up session mainly involves warming up through simple joint activities and brisk walking, gradually bringing the body into a state of exercise. The MICE session mainly consists of continuous jogging, and it is not recommended to take long breaks in the middle. Short adjustments for breathing can be made as needed. The relaxation session mainly involves static stretching of the whole body to relax the muscles and relieve muscle soreness after exercise. The exercise cycle of the MICE group is 12 weeks, with 3 exercise sessions per week, scheduled for Tuesdays, Thursdays, and Saturdays. The purpose of this schedule is to ensure that adolescents have sufficient rest and recovery time, preventing potential sports fatigue caused by daily exercise.

#### 2.2.3 Control group

The control group maintained their normal living and studying status during the experiment period and did not receive additional regular exercise interventions. They carried out their daily studies according to the school’s curriculum arrangement, participated in the recess activities and physical education classes organized by the school. However, the content and intensity of the physical education classes followed the school’s regular teaching plan, and no specialized sports training was conducted. During their spare time, the teenagers in the control group arranged their activities freely and could engage in moderate leisure activities, such as walking, cycling, etc. However, the activity time and intensity were not controlled by the experiment to ensure the naturalness of their daily activities. Regular follow-ups were conducted on the teenagers in the control group to understand their physical conditions and living situations, ensuring that they did not participate in other organized physical exercises or sports intervention programs during the experiment period.

### 2.3 Variables and tools

#### 2.3.1 Executive function

Referring to relevant studies (Akatsuka et al., 2015; Chen et al., 2014), in this study, the GO/NOGO task, the 2-back task, and the More-odd shifting task were selected to test the participants’ inhibitory control, working memory, and cognitive flexibility, respectively. All the above procedures were programmed using E-prime 2.0 and installed on a laptop. Before the test, trained testers explained the tasks to the participants. After each participant completed one test, they signaled the tester, and the tester helped switch to the next test task. The test order was GO/NONO, 2-back, and More-odd shifting.

##### 2.3.1.1 GO/NOGO

In this experimental task, a series of numbers within the range of 0 ~ 9 will be randomly presented in the center of the screen. Among them, any number other than the number “3” is used as the GO stimulus, while the number “3” serves as the NOGO stimulus. After the experiment starts, if the subject sees a number other than “3” presented on the screen, they need to quickly and accurately press the “F” key. If the number presented is “3,” they should refrain from pressing any key. If no key is pressed, the digital image on the screen will automatically disappear after 2000 milliseconds, and then the next trial will commence. Before the formal experiment begins, the participants will first conduct a practice session. The practice session consists of 12 trials, among which the GO trials occur 9 times and the NOGO trials occur 3 times. Once the participants understand the experimental rules, they can start the practice. If the participants still have questions after the practice session, they can press the “Q” key to continue the practice. If they confirm that there are no problems, they can press the “Space” key to start the formal experiment. The formal experiment has a total of 100 trials, with the Go trials presented 75 times and the NOGO trials presented 25 times, and the ratio of their occurrences is 3:1. This test uses the average reaction time and the accuracy rate under the NOGO condition as the performance indicators. Generally speaking, the higher the accuracy and the shorter the reaction time, the better the inhibitory function of the participants.

##### 2.3.1.2 2-back

The experiment uses six English letters “Q, W, E, R, T, G” as visual stimulus materials. Through constructing a specific cognitive task paradigm, it systematically examines the working memory updating function of the participants. A single stimulus letter is centrally displayed on a white background screen in black Song typeface with a font size of 36. The presentation duration is fixed at 2 s, and then it enters a 3-s stimulus onset asynchrony (SOA) stage, during which the screen is in a blank state. The participants need to maintain a high level of concentration and instantly judge whether the currently presented letter is consistent with the second-to-last letter in the sequence. If they are the same, the participants need to quickly press the “F” key on the keyboard; if they are different, they should press the “J” key. The formal experiment is divided into two sections, and each section contains 25 valid trials. To ensure that the participants are familiar with the task rules, 20 practice trials are set before the formal experiment. Timely feedback on correct and incorrect responses is provided during the practice stage, while the feedback function is turned off during the formal experiment. The accuracy rate and the average reaction time are used as the core evaluation indicators. The accuracy rate is the percentage of the number of correct responses to the total number of trials, and the average reaction time is the average time consumed for all correct responses. The two indicators are complementary to each other. A higher accuracy rate and a shorter reaction time jointly indicate a better working memory updating ability of an individual.

##### 2.3.1.3 More-odd shifting

After the experiment starts, numbers ranging from 1 to 9 (excluding 5) will be presented one by one at the center of the computer screen. Each number is displayed in a 36-point Song typeface in black or green for 2000 milliseconds, and then there is an interval of 3 s (stimulus onset asynchrony, SOA) before the next stimulus appears. The experiment consists of three parts of tasks. In the first part, black numbers are presented, and participants are required to judge the magnitude relationship between the presented number and “5.” If the number is greater than “5,” they should press the “J” key, and if it is less than “5,” they should press the “F” key. In the second part, green numbers are shown, and participants need to judge the parity of the numbers. When the number is odd, they should press the “J” key, and when it is even, they should press the “F” key. In the third part, the tasks of the previous two parts are integrated. Participants are required to switch the judgment rules according to the color of the numbers. That is, when the number is black, they should judge its magnitude, and when the number is green, they should judge its parity. The formal test is divided into three sections in the order of A, B, and C. Sections A and B each involve 16 single judgments (without the need for switching), and section C contains 32 judgments, among which 16 involve the process of switching. Finally, the overall accuracy rate, as well as the difference between the average reaction times under the switching condition and the non-switching condition, is used as the evaluation indicators for the cognitive switching function. The smaller the difference and the higher the accuracy, the stronger the participants’ cognitive switching ability.

#### 2.3.2 Demographic information

In this study, a self-developed questionnaire was used to survey the participants’ basic information, including age, gender, socioeconomic status (SES), medical history of diseases (cardiovascular diseases, respiratory diseases, nervous system diseases), contraindications in sports medicine, as well as eyesight. For SES, the MacArthur Scale of Subjective Social Status was adopted to assess the socioeconomic status of adolescents. This scale has been proven to have high reliability and validity. The scale consists of a simple ladder graphic. The ladder has 10 rungs, which are labeled with numbers from 1 to 10, respectively. Number 1 represents the lowest status, and number 10 represents the highest status. Participants were asked to select the rung on the ladder that best represents their subjective socioeconomic status according to their own situations. In addition, in this study, participants were required to fill in whether they had a medical history of major diseases such as cardiovascular diseases, respiratory diseases or nervous system diseases, whether there were any contraindications in sports medicine such as unrecovered joint injuries or severe musculoskeletal diseases, and whether their eyesight was normal, based on their actual situations. The parents of the participants were required to check and sign to confirm the information filled in by the participants, so as to ensure the accuracy of the filled information.

#### 2.3.3 Height and weight

In this study, a standard electronic height and weight measuring instrument (Kangwa WS-RT-3B) was used to measure the height and weight of the participants. Before the measurement, the height and weight measuring instrument was inspected to ensure that its scale was accurate and clear. The measuring instrument was placed on a horizontal and hard ground. The subjects took off their hats, shoes, and thick clothes and stood on the base plate of the height measuring instrument. The heels of their feet were put together, the toes were separated at an angle of about 60 degrees, their bodies were kept straight, their eyes looked straight ahead, and their chins were slightly tucked in. Their arms hung naturally and were close to the sides of their bodies. When the subjects were properly positioned, the instrument would automatically sense and display the height value. To ensure the accuracy of the measurement results, the measurement was repeated twice, and the average value was taken as the final height measurement result. In addition, in this study, the specific BMI values of the participants were calculated according to the BMI calculation formula (BMI = kg/m^2^).

#### 2.3.4 Heart rate monitoring

The heart rates of the HIIE group and the MICE group were monitored to ensure that their exercise loads reached the intervals specified in the research plan. In this study, the Polar Team 2 Team Heart Rate Monitoring System was used to monitor the heart rates of each group during each session. The core component, the heart rate transmitter, is paired with the WearLink+ chest strap. Through the principle of electrocardiogram signals, it can collect heart rates almost in real time, and within a barrier-free range of 150 meters, it can stably transmit the data to the platform of the monitoring system. During the exercise process, the coach can directly view the heart rate change curves of each adolescent. When the monitored heart rate exceeds the preset upper limit of the maximum heart rate, the system will immediately give a prompt and send out an alarm. At the same time, the coach will verbally remind the adolescents to appropriately reduce the exercise intensity or increase the rest time. If the heart rate continuously remains below the lower limit of the target range, the coach will encourage the adolescents to gradually increase the exercise intensity to ensure that the training effect meets the expected goals.

### 2.4 Mathematical statistics

In this study, the statistical analysis was conducted using the SPSS 26.0 software (IBM, New York, NY, United States). For continuous variables, descriptive statistics were carried out using the mean (*M*) and standard deviation (*SD*); for categorical variables, descriptive statistics were performed using frequencies and percentages. Firstly, the Shapiro–Wilk test was combined with the P–P plot and Q–Q plot to conduct the normality test of the data. It was found that the continuous variables approximately presented a normal distribution. Therefore, One-way ANOVA was used for the between-group comparative analysis of the pre-test data. Secondly, ANCOVA was employed to analyze and compare the impact of HIIE and MICT interventions on executive functions and BMI among obese adolescents with low SES. In all statistical analyses, a significance level of *p* < 0.05 was set as the criterion for statistical significance of the differences, and *p* < 0.01 was set as the criterion for highly statistical significance of the differences.

## 3 Results

### 3.1 The basic information of the participants

There were no significant differences in age, gender composition ratio, SES, and BMI among the HIIE group, MICE group, and the control group (*p* > 0.05). The basic information of the three groups is detailed in Table 1.

**Table 1:** The basic information of the HIIE group, the MICE group and the control group.

Variables	HIIE (*n* = 25)	MICE (*n* = 25)	CG (*n* = 25)
Age	13.48 ± 1.12	13.80 ± 1.87	13.44 ± 1.59
Gender (F%)	48.00%	48.00%	52.00%
SES	3.00 ± 1.44	3.00 ± 1.38	3.03 ± 1.43
BMI	26.98 ± 1.55	27.30 ± 1.57	27.17 ± 1.62

F% indicates the proportion of female participants; CG stands for the control group.

### 3.2 Intergroup comparative analysis of executive function before intervention

The results (Table 2) of the intergroup comparative analysis of executive function among the HIIE group, MICE group, and the control group before the intervention show that there were no significant differences in reaction time and accuracy among the three groups in the GO/NO, 2-back, and More odd-shifting tasks (*p* > 0.05). Therefore, the executive functions of the participants in each group were at a roughly similar level before the intervention, which ensures the accuracy of the research results.

**Table 2:** The results of the intergroup comparative analysis of executive function among the HIIE group, MICE group, and the control group before the intervention.

Variables	HIIE (*n* = 25)	MICE (*n* = 25)	CG (*n* = 25)	*F*	*P*
GO/NO					
RT	490.07 ± 86.33	456.92 ± 75.10	451.40 ± 73.79	1.770	0.178
ACC	77.80 ± 11.04	83.20 ± 10.84	79.08 ± 11.93	1.568	0.216
2-back					
RT	1133.40 ± 283.97	1148.19 ± 332.61	1128.73 ± 315.20	0.027	0.974
ACC	50.63 ± 12.67	60.97 ± 18.71	57.40 ± 13.24	3.013	0.055
More-odd shifting					
RT	298.68 ± 168.06	204.35 ± 179.70	253.37 ± 158.16	1.951	0.150
ACC	73.17 ± 14.65	75.30 ± 14.26	75.52 ± 12.78	0.218	0.804

CG represents the control group; RT represents reaction time; ACC represents accuracy.

### 3.3 Intergroup comparative analysis of heart rate between the HIIE group and the MICE group

**Figure 1:**
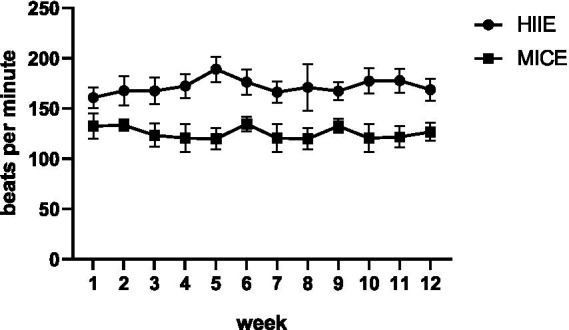
The analysis result of the comparison of the average weekly heart rate between the HIIE group and the MICE group.

During the intervention process, the average heart rate of the HIIE group was (170.88 ± 10.01) beats per minute, and the average heart rate of the MICE group was (130.56 ± 9.06) beats per minute. The difference between the two groups was statistically significant (*t* = 14.93, *p* < 0.001). In addition, a comparative analysis of the average weekly heart rates between the HIIE group and the MICE group is shown in Figure 1. The above results indicate that the intervention process strictly adhered to the heart rate monitoring protocol, which can ensure the accuracy of the research results.

### 3.4 Effects of HIIE and MICE on executive function

#### 3.4.1 Effects of HIIE and MICE on inhibitory control

**Figure 2:**
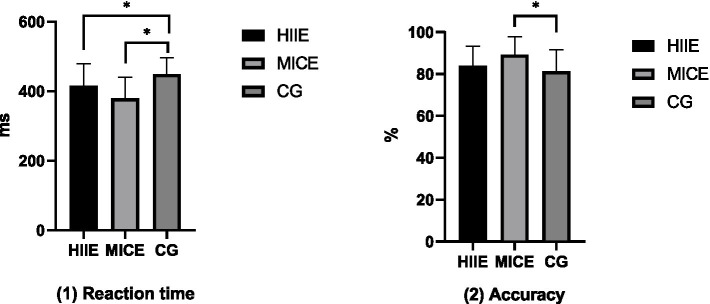
The results of the between-group comparison of inhibitory control between the HIIE group and the MICE group after the intervention. *p < 0.05.

For inhibitory control reaction time, after controlling for relevant confounding factors, the between-subjects effect test revealed a significant main effect of the intervention (*F* = 20.278, *p* < 0.001, *η*^2^ = 0.377). Further pairwise comparison analysis showed that the reaction time of the HIIE group (416.07 ± 62.27 ms) was significantly shorter than that of the control group (449.40 ± 47.49 ms; *p* < 0.001); the reaction time of the MICE group (379.32 ± 60.61 ms) was also significantly shorter than that of the control group (449.40 ± 47.49 ms; *p* < 0.001). However, there was no significant difference in reaction time between the HIIE group and the MICE group (*p* = 0.074). For inhibitory control accuracy, after controlling for relevant confounding factors, the between-subjects effect test revealed a significant main effect of the intervention (*F* = 3.721, *p* = 0.029, *η*^2^ = 0.100). Further pairwise comparison analysis showed that the accuracy of the MICE group (89.27 ± 8.60%) was significantly higher than that of the control group (81.28 ± 12.05%; *p* = 0.009). However, there were no significant differences in accuracy between the HIIE group and either the MICE group or the control group (*p* = 0.074). The detailed results of the between-group comparisons for the HIIE and MICE groups after the intervention are shown in Figure 2.

#### 3.4.2 Effects of HIIE and MICE on working memory

**Figure 3:**
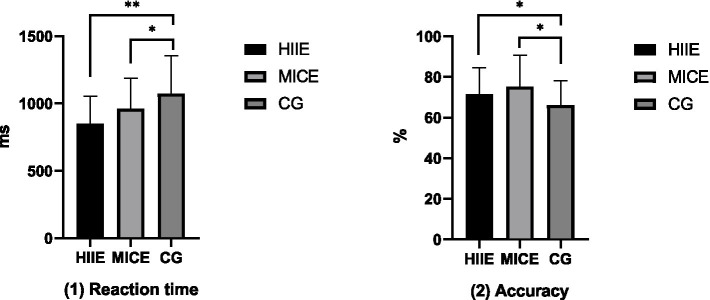
The results of the between-group comparison of working memory between the HIIE group and the MICE group after the intervention. *p < 0.05, **p < 0.01.

For the reaction time of working memory, after controlling for relevant confounding factors, the between-subjects effect of the intervention was significant (*F* = 7.133, *p* = 0.002, *η*^2^ = 0.002). Further paired comparison analysis revealed that the reaction times of the HIIE group (851.40 ± 202.60 ms) and the MICE group (962.19 ± 225.76 ms) were significantly shorter than that of the control group (1074.73 ± 280.81 ms; *p* < 0.05). For the accuracy rate of working memory, after controlling for relevant confounding factors, the between-subjects effect of the intervention was significant (*F* = 4.272, *p* = 0.018, *η*^2^ = 0.113). Further paired comparison analysis showed that the accuracy rates of the HIIE group (71.43 ± 13.00%) and the MICE groups (75.32 ± 15.28%) were significantly higher than that of the control group (66.20 ± 11.98%; *p* < 0.05). The results of the between-group comparison analysis between the HIIE and MICE groups after the intervention are shown in Figure 3.

#### 3.4.3 Effects of HIIE and MICE on cognitive flexibility

**Figure 4:**
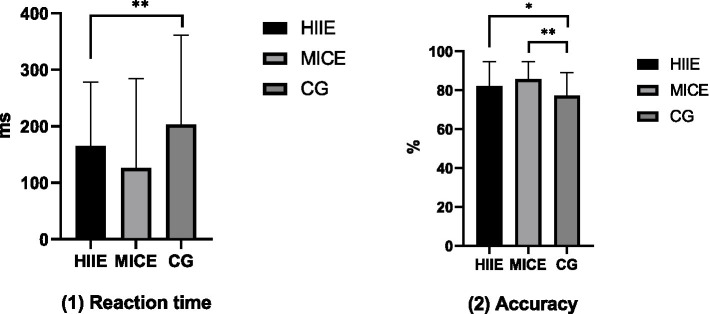
The results of the between-group comparison of cognitive flexibility between the HIIE group and the MICE group after the intervention. *p < 0.05, **p < 0.01.

For the reaction time of cognitive flexibility, after controlling for relevant confounding factors, the between-subjects effect of the intervention was significant (*F* = 5.343, *p* = 0.007, *η*^2^ = 0.138). Further pairwise comparison analysis revealed that the reaction time of the HIIE group (164.68 ± 113.97 ms) was significantly shorter than that of the control group (203.37 ± 158.16 ms; *p* = 0.02). However, there was no statistically significant difference between the MICE group and the control group (*p* > 0.05). For the accuracy rate of cognitive flexibility, after controlling for relevant confounding factors, the between-subjects effect of the intervention was significant (*F* = 5.341, *p* = 0.007, *η*^2^ = 0.138). Further pairwise comparison analysis showed that the accuracy rates of the HIIE group (82.13 ± 12.58%) and the MICE groups (85.73 ± 8.98%) were significantly higher than that of the control group (77.21 ± 11.69%; *p* < 0.05). The results of the between-group comparison analysis between the HIIE group and the MICE group after the intervention are shown in Figure 4.

### 3.5 Effects of HIIE and MICE on BMI

**Figure 5:**
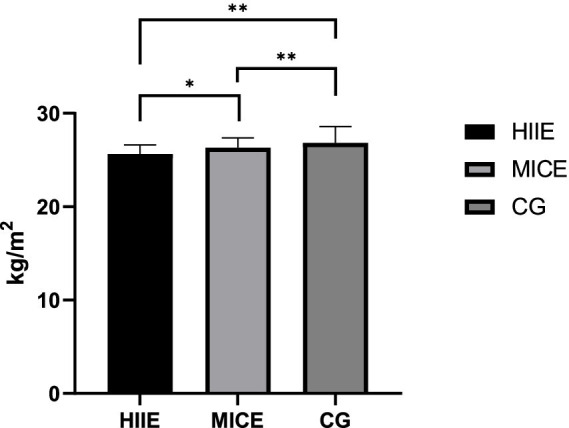
The results of the between-group comparison of BMI between the HIIE group and the MICE group after the intervention. *p < 0.05; **p < 0.01.

For BMI, after controlling for relevant confounding factors, the between-subjects main effect of the intervention was significant (*F* = 12.447, *p* < 0.001, *η*^2^ = 0.260). Further pairwise comparisons showed that BMI in the HIIE group (25.65 ± 0.96) was significantly lower than that in the MICE group (26.32 ± 1.03; *p* = 0.027) and the control group (26.83 ± 1.75; *p* < 0.001). BMI in the MICE group (26.32 ± 1.03) was significantly lower than that in the control group (26.83 ± 1.75; *p* = 0.008). The results of between-group comparisons between the HIIE group and MICE group after intervention are presented in Figure 5.

## 4 Discussion

This study explored the effects of HIIE and MICE interventions on the executive functions of obese adolescents from low SES backgrounds. It was found that both HIIE and MICE can improve the inhibitory control, working memory, and cognitive flexibility of obese adolescents to varying degrees, but there are differences in specific effects. This provides a basis for diverse choices in improving the executive functions of obese adolescents from low SES backgrounds through exercise interventions.

### 4.1 HIIE can effectively promote the development of executive function

The results of this study show that HIIE can effectively promote the development of executive function in obese adolescents from low SES backgrounds, especially in terms of reaction time of inhibitory control, reaction time and accuracy of working memory, as well as reaction time and accuracy of cognitive flexibility. This finding further supports previous studies (Reyes-Amigo et al., 2022; Tottori et al., 2019), indicating that HIIE has a positive effect on the executive function of adolescents. The positive impact of HIIE on the executive function of obese adolescents from low SES backgrounds is attributed to multiple factors, including the improvement of cardiovascular function, the regulation of brain-derived neurotrophic factor (BDNF), and the enhancement of neural plasticity.

The results of this study show that the average heart rate of the HIIE group is relatively high, which means a stronger stimulation to the cardiovascular system, and thus can promote the development of the cardiopulmonary function of adolescents (Nagashima et al., 2010). Related studies (Birkett et al., 2019; Neuendorf et al., 2023) have found that after HIIE training, the individual’s maximal oxygen uptake (VO₂max) increases significantly, which means that the body can absorb and utilize more oxygen, providing a more sufficient energy supply for the brain. When the brain receives sufficient oxygen and nutrients, the metabolic activities of neurons become more active, and the synthesis and release of neurotransmitters are also more stable, thus helping to improve executive function (Ogoh, 2017; Poels et al., 2008). In addition, BDNF plays a crucial role in the intervention of HIIE on executive function. Relevant studies (Li et al., 2022; Rentería et al., 2020) have found that HIIE can significantly increase the expression and secretion of BDNF. After BDNF enters the brain through the bloodstream, it binds to the receptors on the surface of neurons, activates downstream signaling molecules, promotes the proliferation, survival, and differentiation of neurons, enhances synaptic plasticity, and thus promotes the development of executive function (Kowiański et al., 2018; Lu et al., 2023).

The intense exercise stimuli during HIIE can prompt the brain to undergo a series of adaptive changes. At the neuronal level, HIIE can increase the dendritic branches and spine density of neurons, thereby expanding the information transmission network among neurons (Dos Santos et al., 2020; Li et al., 2020). Some studies (Dos Santos et al., 2020; Li et al., 2020) have found that after a period of HIIE training, the dendrites of neurons in brain regions closely related to executive function, such as the hippocampus and prefrontal cortex of experimental animals, become more complex. This implies that more synaptic connections can be established among neurons, improving the efficiency and accuracy of information transmission. In addition, the realization of executive function depends on the coordinated work of multiple brain regions, such as the prefrontal cortex, parietal cortex, and hippocampus (Aron, 2008). And HIIE can enhance the neural connections among these brain regions, enabling them to conduct information exchange and collaboration more effectively when performing tasks (Calverley et al., 2020).

### 4.2 MICE can effectively promote the development of executive function

The results of this study found that MICE can effectively improve the executive function of obese adolescents with low SES, especially reflected in the response time and accuracy rate of inhibitory control and working memory, as well as the accuracy rate of cognitive flexibility. This result is consistent with the findings of previous studies (Liu et al., 2020). Regarding the explanation of its internal causes, it can be explained from the aspects of stable cardiopulmonary function stimulation and metabolic regulation.

MICE is carried out at a relatively stable moderate intensity, keeping the body in an aerobic metabolism state for a long time. During the exercise, the stroke volume of the heart gradually increases, which can more effectively deliver oxygen-rich blood to the brain (Lee et al., 2023). This, in turn, enhances the metabolic activities of neurons, thus providing a good physiological foundation for the development of executive functions (Ogoh, 2017; Poels et al., 2008). In addition, MICE can also promote vascular health, enhance vascular elasticity, reduce vascular resistance, and improve the blood circulation in the brain, ensuring that all areas of the brain can receive sufficient nutrients and oxygen, and thus having a positive impact on executive functions (Oliva et al., 2023; Ramos et al., 2015).

The MICE can regulate the energy metabolism and material metabolism of obese adolescents. In terms of energy metabolism, MICE can increase the body’s energy consumption, promote the oxidation and decomposition of fat, help obese adolescents lose weight, and reduce body fat content (Nassis et al., 2005; Rowlands et al., 1999). Relevant studies (Laurent et al., 2020; Mamrot and Hanć, 2019) shows that the improvement of body composition is conducive to the development of adolescents’ executive function. In terms of material metabolism, MICE can promote the synthesis and release of neurotransmitters such as dopamine and serotonin, thereby promoting the development of executive functions (Tsai et al., 2024).

### 4.3 The differences in the intervention effects between HIIE and MICE

Overall, both HIIE and MICE have positive effects on different dimensions of executive function, namely inhibitory control, working memory, and cognitive flexibility. However, there are differences in specific effects. Among them, MICE has a better intervention effect on the accuracy rate of inhibitory control, while HIIE has a better intervention effect on the reaction time of cognitive flexibility. This provides a basis for diverse choices for improving executive function through exercise intervention.

The results of this study are similar to previous research, all indicating that HIIE and MICE have a selective promoting effect on executive function. However, there are still inconsistencies when it comes to specific related indicators. For example, a systematic review and meta-analysis (Wu et al., 2023) comparing the effects of HIIE and MICE interventions on the inhibitory control of healthy populations found that there was no significant difference in the intervention effects between MICE and HIIE. Jia (2022) explored the effects of HIIE and MICE interventions on the executive function of obese adolescents and also found that both HIIE and MICE could improve the executive function of obese adolescents, but the effect of MICE intervention on working memory was better than that of HIIE. In addition, exercise adherence may be a potential factor contributing to the differences in the effects of the two exercise modes. Although in this study, the adolescents in both the HIIE group and the MICE group were able to complete the training tasks well, the high-intensity exercise phase of HIIE is somewhat challenging, and it also brings more a sense of accomplishment and freshness, which may increase their exercise motivation and adherence. In contrast, the exercise intensity of MICT is relatively low, and the exercise process is relatively monotonous, which may make some adolescents feel bored during the training process, thus affecting their exercise adherence. Higher exercise adherence means that adolescents can participate in the training more actively and better achieve the training goals. Therefore, they may obtain more positive exercise effects in the more complex executive control task of cognitive flexibility.

### 4.4 HIIE and MICE both exert beneficial intervention effects on BMI

The results of this study showed that both HIIE and MICE exerted positive improving effects on participants’ BMI, with HIIE yielding superior intervention outcomes. This finding is consistent with the core consensus in the existing field of exercise intervention, confirming the effectiveness of physical exercise in improving weight-related indicators (Abdelbasset et al., 2020; Gremeaux et al., 2012). The more favorable intervention effect of HIIE may be attributed to its unique physiological mechanism. In contrast to the stable and continuous energy expenditure of MICE, HIIE generates higher instantaneous energy consumption during exercise and triggers a more significant excess post-exercise oxygen consumption effect, enabling the body to maintain a high metabolic level even after exercise and prolonging the duration of fat oxidation for energy supply (Panissa et al., 2021). Additionally, HIIE features shorter exercise duration and higher time efficiency, which is more conducive to enhancing participants’ long-term exercise adherence and lays a solid foundation for the sustained improvement of BMI (Tschakert and Hofmann, 2013). This is also consistent with the conclusions of previous studies that HIIE has greater advantages in weight management, further verifying its potential as a preferred exercise modality for BMI intervention (Wewege et al., 2017). Furthermore, these findings provide empirical support for the formulation of individualized exercise intervention programs in the future, namely that HIIE can be prioritized for populations requiring efficient BMI improvement.

### 4.5 The practical significance and application value of this study

The results of this study have important guiding roles in the formulation of exercise intervention programs for obese adolescents from low SES backgrounds, school physical education, and community health promotion.

In terms of formulating exercise intervention programs, the research findings provide a scientific basis for precisely formulating exercise intervention programs for obese adolescents from low SES backgrounds. The research results suggest that when formulating exercise intervention programs, the exercise methods can be selectively chosen in a targeted manner according to factors such as the physical conditions, hobbies, and exercise goals of adolescents. For adolescents with better physical fitness, a stronger sports foundation, and a desire to significantly improve their executive function in the short term, HIIE can be preferentially recommended; while for adolescents with relatively weaker physical fitness, those who are just starting to exercise, or those who prefer a steady improvement in their health level, MICE may be a more suitable choice.

In terms of school physical education, it provides a reference for the setting of school physical education curriculums and the improvement of teaching methods. When designing physical education curriculums, schools can add teaching contents of HIIE and MICE to enrich the diversity of physical education curriculums and meet the exercise needs of different students. In physical education teaching, a combination of HIIE and MICE can be adopted, enabling students to experience the exercise effects of different exercise intensities in a single physical education class. In addition, teachers can conduct stratified teaching for students according to the results of their executive function tests. For students with relatively weaker executive function, more attention and guidance can be given to help them improve their executive function through exercise.

In terms of community health promotion, it helps communities carry out more targeted adolescent health promotion activities. Communities can formulate health promotion plans suitable for obese adolescents from low SES backgrounds according to the research results, and organize regular HIIE or MICE exercise activities. Through the active participation and promotion of the community, a good exercise atmosphere can be created to promote the healthy growth of obese adolescents from low SES backgrounds.

### 4.6 The limitations of this study

Firstly, this study has certain limitations in terms of sample size. Although 120 obese adolescents from low SES backgrounds were selected as participants in the study, from a statistical perspective and considering practical promotion and application, the sample size is relatively limited. Among the group of obese adolescents from low SES backgrounds, there may be significant individual differences due to different regions, family backgrounds, and living environments. A relatively small sample size may not comprehensively cover these differences, resulting in insufficient representativeness of the research results and having a certain impact on the universality and reliability of the research conclusions. Future research can further expand the sample size to include obese adolescents from low SES backgrounds in different regions and living environments, so as to improve the representativeness and promotional value of the research results.

Secondly, in terms of the experimental period, the exercise intervention period of this study is 12 weeks, which is relatively short. Although significant improvements in the executive function of obese adolescents from low SES backgrounds were observed after 12 weeks of HIIE and MICE, the long-term effects of exercise on executive function remain unclear. The development and improvement of executive function is a long-term process. A short-term exercise intervention may only initiate the relevant physiological and psychological mechanisms. The continuous promoting effect of long-term persistent exercise on executive function, as well as the maintenance of executive function after the cessation of exercise, still requires further research. Subsequent research can extend the experimental period, set different time points for measurement, and observe the long-term dynamic changes in the impact of exercise on executive function.

Finally, the research design did not include a dietary intervention-related module, resulting in incomplete variable control. Focusing on comparing the intervention effects of the two exercise modes, this study did not systematically assess or control the participants’ dietary status, nor did it set up a combined diet + exercise intervention group. Consequently, it was unable to quantify the regulatory role of dietary factors in the effectiveness of exercise interventions. Therefore, this study suggests that subsequent research may incorporate dietary assessment and intervention components. On one hand, systematic collection of participants’ dietary data through dietary diaries, food frequency questionnaires, and other methods can be used to analyze the regulatory effect of dietary patterns on exercise intervention outcomes. On the other hand, separate exercise groups, dietary intervention groups, and combined diet + exercise intervention groups can be established to compare the improvement effects of different intervention modes on executive function, BMI, and related physiological indicators.

## 5 Conclusion

Through a 12-week intervention of HIIE and MICE on obese adolescents from low SES backgrounds, this study systematically explored the impacts of these two exercise methods on their executive function. It was found that both HIIE and MICE could significantly improve the executive function of obese adolescents from low SES backgrounds, but there was a slight selective promoting effect between the two. Additionally, both HIIE and MICE can effectively reduce participants’ BMI, with the former showing superior intervention efficacy. This study plays a positive and promoting role in the formulation of exercise programs for obese adolescents from low SES backgrounds, the reform of school physical education teaching, and the construction of community sports.

## Data Availability

The raw data supporting the conclusions of this article will be made available by the authors, without undue reservation.

## Footnotes

1
http://123.5ikfc.com/bmi/child.html
